# A False Accusation

**Published:** 1881-11

**Authors:** 


### A FALSE ACCUSATION.

*The Homœopathic Physician:* A Monthly Journal of Medical Science. Philadelphia.

THIS recent addition to the periodical literature of homœopathy is, we believe, intended to supply the void created by the sudden demise of the Anglo-American Journal, called *The Organon.* Its articles are of much the same quality, *its sneers at all physicians who do not believe in the marvellous efficacy of C. M.'s fully as contemptuous as were those of its predecessor.*

Of the medical men who took part in the International Homœopathic Convention the modest editor says, they "have never practiced homœopathically," and, "for the most part," they "know nothing of the homœopathy of Hahnemann." The moral they seek to derive from the proceedings is "the great necessity for the *International Hahnemannian Association.*"

English practitioners who like literature of this type may be

---

man who says this, is wholly ignorant of my habits of reading, and equally so as to any *one* book I have or have not read. And yet this does not prevent his very serious accusation of neglect as to an important branch of professional reading. Whether stupidity or impudence predominate in this accusation it is not very material that we should decide.

From Hahnemann's time till now we have been compelled to listen to his "lack of pathology," and to the opposition to this science on the part of his followers. The din of this has been as constant and monotonous as the drone of the bagpipe, and quite as meaningless. On page 461 *et seq.*, vol. 1, of THE HOMŒOPATHIC PHYSICIAN, the Doctor may find a distinction made between *true* pathology and the *pseudo*. It is not our fault if he finds his hypotheses, by this just discrimination, relegated to the latter category.

interested in hearing that Mr. Heath, of Ebury street, is the agent for its sale.—*M. H. Review.*

[Will the *M. H. Review* kindly *quote* passages showing that THE HOMŒOPATHIC PHYSICIAN "sneers at all physicians who do not believe in the marvellous efficacy of C. M.'s." ? Prove your accusations, gentlemen !]

